# Effects of dietary macronutrient distribution on resting and post-exercise metabolism

**DOI:** 10.1186/1550-2783-11-S1-P6

**Published:** 2014-12-01

**Authors:** Eric T Trexler, Abbie E Smith-Ryan, Hailee L Wingfield, Malia N Melvin, Erica J Roelofs

**Affiliations:** 1 Applied Physiology Laboratory, The University of North Carolina, Chapel Hill, North Carolina, USA

## Background

Previous research has demonstrated that habitual dietary macronutrient distribution affects energy substrate utilization at rest and during exercise. The primary purpose of the current study was to examine the relationships between habitual baseline macronutrient intakes, expressed relative to bodyweight and percentage of total energy intake, and metabolism at rest and after exercise in women.

## Methods

Twenty recreationally active women (Mean ± SD; Age 24.6 ± 3.9 yrs; Height 164.4 ± 6.6 cm; Weight 62.7 ± 6.6 kg; BF% 28.2 ± 4.8%) volunteered for the current study. Prior to metabolic testing, participants completed strength testing to determine their 1RM for six resistance exercises and completed a 3-day dietary log. Participants were provided with detailed instructions for accurately logging food intake and portion sizes, and instructed to record their regular food intake on two week days and one weekend day. Logs were analyzed using The Food Processor software (ESHA Research, Salem, OR, USA). Body composition was determined using dual-energy X-ray absorptiometry (DXA). Exercise was prescribed using baseline strength data and heart rate reserve calculations. Respiratory exchange ratio (RER) and resting energy expenditure (REE) were analyzed via indirect calorimetry (Parvomedics TrueOne 2400) before exercise (PRE), and during minutes 0-10 (IP), 25-35 (30min), and 50-60 (60min) post-exercise. Bivariate correlations and independent samples t-tests were completed using SPSS software (Version 19.0; IBM, Somers, NY, USA). Participants were stratified based on fat intake as a percentage of total caloric intake (high fat > 35% of total kcals, n=11; low fat < 35% of total kcals, n=9) and relative to bodyweight (high fat > 1.3 g/kg, n=11; low fat < 1.3 g/kg, n=9). Consent to publish the results was obtained from all participants.

## Results

A significant inverse relationship between carbohydrate (CHO) intake (g/kg) and REE was observed at IP (r=-0.539, p=0.014), 30min (r=-0.569, p=0.009), and 60min (r=-0.577, p=0.008), with a trend toward significance at PRE (r=-0.413, p=0.07). Participants consuming > 35% of kilocalories from fat had a significantly higher bench press 1RM (p=0.048) and lower RER at 30min (p=0.033) and 60 min (p=0.033). Participants consuming > 1.3 g/kg of fat per day trended toward lower fat mass (p=0.09) and BF% (p=0.07).

## Conclusions

Results indicate that baseline macronutrient distribution impacts post-exercise energy metabolism in women. Higher fat and lower CHO intake was associated with greater post-exercise REE and lower post-exercise RER, indicating greater fat utilization. Higher fat intakes may also influence resistance exercise performance and body composition; participants with higher fat intake had a greater bench press 1RM and trended towards lower indices of adiposity. These results suggest that the relative contributions of CHO and fat to total caloric intake may have significant implications for metabolism and body composition in women. Future research should evaluate the effects of modulating habitual macronutrient distribution to impart favorable effects on energy metabolism, body composition, and resistance exercise performance in women.

